# Editorial: New perspectives of L2 acquisition related to human-computer interaction (HCI)

**DOI:** 10.3389/fpsyg.2022.1098208

**Published:** 2022-11-25

**Authors:** Fanny Meunier, Marcel Pikhart, Blanka Klimova

**Affiliations:** ^1^Institute for Language and Communication, Université catholique de Louvain, Louvain-la-Neuve, Belgium; ^2^Faculty of Informatics and Management, University of Hradec Králové, Hradec Kralove, Czechia

**Keywords:** L2 acquisition, FLL, SLA, EFL, ESL, human computer interaction

The present Research Topic aimed to collect cutting-edge research into L2 acquisition with respect to very recent findings in psycholinguistics, cognitive science, artificial intelligence (AI), Information and Communication Technologies (ICT), data science, and other human-computer interaction (HCI) related areas. Unprecedented developments and trends in AI and ICT need our attention as they (will) dramatically influence how languages are acquired and used. The four research articles published under the topic cover a range of areas on which international teams are working worldwide. They pay tribute to the value of interdisciplinarity as a key to better understanding the issues at play when technology meets language, always keeping in mind the needs and best interests of the human end users. The methodologies adopted range from experimental to qualitative studies; the target populations range from young children to English for specific purposes learners; and the language foci cover pronunciation, vocabulary, sentence comprehension, and also emotions more generally.

Song et al. address interactions between voice-activated AI assistants and human speakers; they also discuss the implications of such interactions for Second Language Acquisition. Their experimental study shows that whilst voice-activated artificially intelligent (voice-AI) assistants are very effective at processing spoken commands by native speakers, the results are much less good when the commands are produced by L2 speakers. Their study focuses on minimal vowel pairs and on Korean-speaking L2 learners of English. The AI assistant (Alexa in the present case) only achieves a 55% accuracy rate with L2 productions, compared to 98% for native productions. The study also examines modifications following a misrecognition by Alexa in the first attempts and shows that, in such cases, L2 learners made acoustic modifications which exhibited some, but not all, of the predicted characteristics of clear speech and target-like pronunciation. Despite functional fluency in a second language, subtle but significant pronunciation difficulties may persist and impact HCI. When voice-AI does not recognize L2 utterances, previous research has shown that learners tend to abandon their communication attempt. The present study suggests, in contrast, encouraging learners to repeat their utterance rather than abandoning it. Voice-AI can thus also prove useful as a pedagogical tool for learning/improving L2 pronunciation.

Köhler-Dauner et al. use technology to experimentally monitor children's (18–36 months) and mothers' physiological reactions in three play-related episodes: a resting phase, a structured play phase, and a free play situation. Their findings indicate that higher quality of maternal caregiving helps children to regulate themselves effectively (e.g., by contributing to the child's mental and physical wellbeing, which in turn helps him/her balance and regulate negative emotions). Although the study did not focus on language use, such results are worth sharing with language specialists given the role and importance of emotions in language acquisition and use. We believe that studies of that type also encourage educational stakeholders to enhance parental collaboration, support and attention when it comes to early stages of language acquisition, be it for native or additional languages.

Rafiq et al., for their part, offer new qualitative perspectives in HCI for the design of an English language mobile module in science, technology, engineering and mathematics (STEM). In a qualitative study using semi-structured interviews, the authors covered four main themes: the importance of learning English, learners' reported problems, their language learning strategies and their readiness in using a mobile app. Mobile learning seemed an *ad hoc* option for learners. Beyond the typical needs related to app usage (e.g., user-friendliness and comfort), the needs' based approach adopted revealed the importance of vocabulary acquisition in ESP as a primer for all other skills. It also testified to the anxiety that STEM learners face in terms of vocabulary use. In addition, participants also mentioned the importance of audio–visual materials to support vocabulary acquisition and stressed the importance of the teacher's role to scaffold language learning.

Boustani et al.'s study analyzes how multisensory input modulates L2 Sentence Comprehension. A blend of visual, auditory, and kinesthetic/tactile senses (coined as exvolvement) and a combination of auditory, visual, kinesthetic/tactile, olfactory, and gustatory modalities (coined as involvement) were used. The authors focus on two specific measures of the event-related potential (ERP) tool to measure brain response as a result of specific sensory, cognitive, or motor events: N400 and P200. The former is sensitive to prediction and expectation functions, as well as semantic processing of words and sentences, whilst the latter is sensitive to diverse language-oriented stimuli, with functions more directed toward attention. Using various multisensory input (to present a list of unfamiliar L2 words subsequently embedded in an acceptability judgment task with 360 pragmatically correct and incorrect sentences), the authors found that the combination of five senses leads to more accurate and quicker responses, thereby empowering the subjects' performance on the acceptability judgment task. Overall, they concluded that the more senses are involved in learning a new concept, the more probable the new information is committed to long-term memory and less susceptible to forgetting.

All in all, despite their varied foci, results and methodologies, the four studies invite us not to underestimate the centrality of “humans” in human-computer interactions. Whilst technology is increasingly used in language learning and teaching, human interactions, teacher scaffolding, emotions, and multisensory aspects are more central than ever and should never become the “parent pauvre” of language learning and teaching.

## Author contributions

All authors have read and agreed with the final version of the editorial.

## Funding

The research is part of the Language in the Human-Machine Era (LITHME) project.

## Conflict of interest

The authors declare that the research was conducted in the absence of any commercial or financial relationships that could be construed as a potential conflict of interest.

## Publisher's note

All claims expressed in this article are solely those of the authors and do not necessarily represent those of their affiliated organizations, or those of the publisher, the editors and the reviewers. Any product that may be evaluated in this article, or claim that may be made by its manufacturer, is not guaranteed or endorsed by the publisher.

